# An Unusual Presentation of Parathyroid Adenoma in an Adolescent: Calcific Achilles Tendinitis

**DOI:** 10.4274/jcrpe.2193

**Published:** 2015-12-03

**Authors:** Selim Kurtoğlu, Leyla Akın, Mustafa Kendirci, Sedat Çağlı, Salih Özgöçmen

**Affiliations:** 1 Erciyes University Faculty of Medicine, Department of Pediatric Endocrinology, Kayseri, Turkey; 2 Erciyes University Faculty of Medicine, Department of Ear Nose and Throat, Kayseri, Turkey; 3 Erciyes University Faculty of Medicine, Department of Rheumatology, Kayseri, Turkey

**Keywords:** Achilles, adolescent, calcific tendinitis, Doppler, hyperparathyroidism, Parathyroid adenoma, tendonopathies

## Abstract

Primary hyperparathyroidism (PHPT) in children and adolescents is a rare condition. PHPT is usually sporadic and caused by parathyroid adenoma. Patients may present with bone pain, proximal myopathy, bony deformities, fractures, renal calculi, mass on the neck, or acute pancreatitis. A sixteen-year-old boy presented to our outpatient clinic with difficulty in walking due to swelling of both ankles. Ultrasonography revealed intratendinous calcific nodules in both Achilles tendons. Serum biochemistry showed hypercalcemia and hypophosphatemia. Serum parathormone level was high (512 pg/mL). Parathyroid scanning revealed a suspected parathyroid adenoma. The patient underwent parathyroidectomy and the diagnosis of parathyroid adenoma was confirmed by histopathology. Serum levels of parathyroid hormone, phosphate, and calcium returned to normal, and the tenderness over the Achilles tendon and the flow pattern on Doppler examination disappeared as well. In conclusion, hyperparathyroidism should be kept in mind in the differential diagnosis of tendonopathies. Early diagnosis can be crucial for prevention of severe complications.

**WHAT IS ALREADY KNOWN ON THIS TOPIC?**

Patients with parathyroid adenoma may present with bone pain, proximal myopathy, bony deformities, fractures, renal calculi, mass on the neck, or acute pancreatitis. In the literature, there is a case report of tendinitis of patellar ligament and quadriceps as the initial presentation of hyperparathyroidism.

**WHAT THIS STUDY ADDS?**

Here, we report a patient with parathyroid adenoma presenting initially with calcific Achilles tendinitis. Reporting the present case, we want to emphasize that parathyroid adenoma should be included in the differential diagnosis of heel pain and calcific tendinitis in adolescents.

## INTRODUCTION

The disorders of the parathyroid glands in pediatric patients are rare conditions with significant morbidity (1). Primary hyperparathyroidism (PHPT) in childhood is seen with an estimated incidence of around 2-5/100,000 (2,3). Because of its rarity, the diagnosis of the disease can be missed by pediatricians until irreversible organ damage has occurred. Patients may present with bone pain, proximal myopathy, bony deformities, fractures, renal calculi, mass in the neck or acute pancreatitis. In children, PHPT presents as a more severe disease than in adults (4,5).

In a literature survey, we have encountered a case report of tendinitis of the patellar ligament and of the quadriceps as the initial presentation of hyperparathyroidism in a young man (6). Reporting the present case, we want to emphasize that parathyroid adenoma should be included in the differential diagnosis of calcific tendinitis of the Achilles in adolescents.

## CASE REPORT

A 16-year-old male presented to our outpatient clinic with complaints of bilateral pain and swelling along his Achilles tendon in the past four weeks. He stated that the pain was usually worse during and after walking. He was a high school student and was not involved in any sport activity. A closer questioning revealed a transient hematuria which had occurred two weeks ago. Medical history was unremarkable otherwise. Family history was also negative for known diseases including cancers.

Physical examination revealed normal findings except for tenderness and swelling in the area where the patient complained of pain. The height of the patient was 172 cm (50th percentile) and his weight was 63 kg (50th percentile). Pubertal stage conformed to Tanner 4 according to Tanner staging. Blood pressure was 110/70 mmHg.

**Figure 1:**
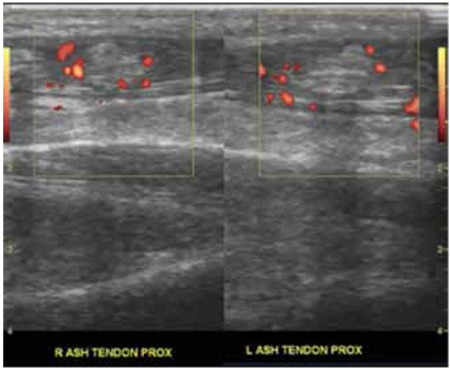
Calcific nodules and surrounding increased Doppler signals localized in both Achilles tendons on the longitudinal Doppler sections

Total blood count was normal. Blood chemistry showed a serum calcium level of 14.4 mg/dL (N: 8.4-10.6 mg/dL), phosphate: 2.4 mg/dL (N: 2.3-4.7 mg/dL), alkaline phosphatase: 245 U/L (N: 40-360 mg/dL), magnesium: 0.8 mmol/L (N: 0.5-1.1 mg/dL), creatinine: 0.8 mg/dL, total protein: 7.7 g/dL, albumin: 4.1 g/dL, alanine aminotransferase: 15 U/L, and aspartate aminotransferase: 19 U/L. Serum glucose and prolactin levels were within normal limits. Urinary calcium/creatinine ratio was 0.2. Parathormone (PTH) level was 512 pg/mL (N: 5-65 pg/mL). Anteroposterior and lateral radiograms of both ankles were normal. A superficial ultrasonography performed using GE logic5P Doppler Sonography device and linear probe (7-12 MHz) revealed calcific nodules and increased Doppler signals surrounding the nodules, which were localized in both Achilles tendons on the longitudinal Doppler sections (Figure 1). Renal ultrasonography was normal. Intravenous saline infusion (3000 mL/day), furosemide (1 mg/kg every six hours) and prednisolone (60 mg/day) were started. Neck ultrasonography revealed a 12x10 mm hypoechoic lesion near the left thyroid lobe, suggesting a parathyroid adenoma. The dual-phase 15 mCi Technetium-99m (99mTc) sestamibi scanning revealed increased activity in the left thyroid lobe, indicating parathyroid adenoma. On the second day of admission, it was observed that the hypercalcemia did not respond to medical treatment and the patient was admitted to pediatric intensive care unit due to severe hypercalcemia (calcium: 18 mg/dL). Serum calcium levels decreased to 13 mg/dL after intravenous calcitonin (0.8 IU/kg/d) was added to the treatment.

A minimally invasive parathyroidectomy was planned. On the day of surgery, serum PTH level was 1222 pg/mL. PTH level was measured intraoperatively 10 minutes after the left superior gland was excised and detected to be decreased more than 50% of the initial PTH level. The left inferior gland was apparently normal. Left superior parathyroid gland was excised guided by gamma probe.

On post-operative day 3, the laboratory data were: calcium: 9.9 mg/dL, phosphate: 3.9 mg/dL, alkaline phosphatase: 176 U/L, and PTH: 12.5 pg/mL. After parathyroidectomy, the swelling also resolved and the patient had some relief from his pain. On the follow-up visit two months after the surgery, the tenderness over the Achilles tendon and the flow pattern on Doppler examination had both disappeared.

## DISCUSSION

Most of the literature on PHPT in children is limited to case reports and small series. Moreover, the age of the patients in these study populations ranges from 0 to 40 which further complicates the understanding of the disease in children (7,8,9,10). Consequently, the recognition and diagnosis of the disease is usually delayed (11). Despite being a rare disease in childhood, PHPT is known to present differently in children than in adults (4,5). In a retrospective study including 15 children and adolescents with PHPT, it was found that clinical features at presentation included bone pain (86.6%), proximal myopathy (46.6%), bony deformities (53.3%), fractures (60%), palpable osteitis fibrosa cystica (33.3%), renal calculi (40%), palpable neck swelling (13.3%), and acute pancreatitis (6.67%) (7).

PHPT is most often sporadic and caused by a parathyroid adenoma. However, it may also occur due to the hyperplasia of the glands, especially as a part of multiple endocrine neoplasia (MEN)-1 and 2 syndromes or familial non-MEN PHPT. Our patient did not develop additional features suggestive of MEN.

There is limited information in the literature regarding the optimal surgical treatment of PHPT in children. In a previous study, Durkin et al (8) showed that PHPT was uniformly caused by single-gland disease in patients younger than 18 years and without a family history. The authors reported that minimally invasive parathyroidectomy with preoperative localization and intraoperative PTH (iPTH) testing was successfully carried out in these patients (8). Similarly, in our patient, the operative procedure consisted of unilateral neck exploration directed by the preoperative localization, iPTH testing, and removal of the left superior parathyroid gland.

The terminology of Achilles tendon pathology used in the medical literature is confusing. The differential diagnosis of posterior heel pain includes mid-portion Achilles tendinopathy, acute or chronic paratendinopathy, retrocalcaneal bursitis, and superficial calcaneal bursitis (12). Damage to the Achilles tendon usually results from chronic overuse by repetitive microtrauma to the tendon. Achilles tendinopathy often presents with soft-tissue swelling, local tenderness, and crepitus. X-rays rarely show calcification of the tendon itself or of the soft tissues surrounding the tendon. In our patient, there was no history of chronic overuse of the Achilles tendon. In the literature, there is a report of a patient, a competitive basketball player, in whom chronic tendinitis secondary to hyperparathyroidism was recognized only after avulsion of the patellar ligament, of the left quadriceps tendon, and of the right triceps tendon (6). In our case, the patient presented with calcific tendinitis just before a severe hypercalcemia developed. Obviously, unless recognized in time, this patient could have presented with a more drastic picture with complications of severe hypercalcemia, such as cardiac arrhythmia.

Reporting this case, we wish to highlight the importance of considering parathyroid adenoma, a rare but treatable disease in childhood, in the differential diagnosis of heel pain. Despite its rarity, the awareness of this disease by clinicians can be crucial in some patients before development of serious and permanent sequelae.
